# Supplementary material for: Anti-Virulence Properties of Coridothymus capitatus Essential Oil against Pseudomonas aeruginosa Clinical Isolates from Cystic Fibrosis Patients
Source: Microorganisms. 2021 Oct 29;9(11):2257. doi: 10.3390/microorganisms9112257 (PMC8623622; doi:10.3390/microorganisms9112257)
Supplement: Supplementary file 1 [file microorganisms-09-02257-s001.zip › microorganisms-1386527-SI.pdf]

microorganisms

MDPI

Article

Anti-Virulence Properties of *Coridothymus capitatus* Essential Oil Against *Pseudomonas aeruginosa* Clinical Isolates from Cystic Fibrosis Patients

Gianluca Vrenna [1,†], Marco Artini [1,†], Rino Ragno [2,3], Michela Relucenti [4], Ersilia Vita Fiscarelli [5], Vanessa Tuccio Guarna Assanti [5], Rosanna Papa [1,*] and Laura Selan [1,*]

[1] Department of Public Health and Infectious Diseases, Sapienza University, p.le Aldo Moro 5, 00185 Rome, Italy; gianluca.vrenna@uniroma1.it (G.V.); marco.artini@uniroma1.it (M.A.)
[2] Department of Drug Chemistry and Technology, Sapienza University, p.le Aldo Moro 5, 00185 Rome, Italy; rino.ragno@uniroma1.it
[3] Rome Center for Molecular Design, Department of Drug Chemistry and Technology, Sapienza University, p.le Aldo Moro 5, 00185 Rome, Italy
[4] Department of Anatomy, Histology, Forensic Medicine and Orthopaedics, Sapienza University of Rome, via Alfonso Borelli 50, 00161Rome, Italy; michela.relucenti@uniroma1.it
[5] Unit Cystic Fibrosis Diagnostic Microbiology and Immunology Diagnostics, Diagnostic Medicine and Laboratory Department, Bambino Gesù Children's Hospital, 00165 Rome, Italy; evita.fiscarelli@opbg.net (E.V.F.); vanessa.tuccio@opbg.net (V.T.G.A.)
* Correspondence: rosanna.papa@uniroma1.it (R.P.); laura.selan@uniroma1.it (L.S.)
† These authors equally contributed to the work

Supplementary materials

Table S1. The eleven *P. aeruginosa* clinical isolates and their characterization by several properties [32].

ID pt	ID	SAM	date	Str	Ph	CAR	PTC	AM	QUIN	MB	CEF	COL	1St	E	L
22	23P	ESP	6/24/2005	PA MDR MBL+	sm	R	S	R	R	S	R	S		X	
24	26P	TF	8/27/2008	PA	i	S	S	S	S	I	S	S	X		
24	27P	AT	1/31/2017	PA	sm	S	S	S	S	S	S	S			X
25	28P	ESP	5/24/2012	PA	sm	S	S	S	S	S	S	S	X		
25	29P	AT	9/13/2017	PA	m	S	S	S	S	S	S	S			X
9	30P	ESP	9/6/2010	PA	i	S	S	S	S	I	S	S	X		
9	31P	ESP	1/11/2017	PA	m	S	S	S	R	S	S	S			X
26	32P	AT	12/5/2006	PA	sm	S	S	R	S	I	S	S	X		
26	33P	AT	12/28/2016	PA	m	S	S	S	S	I	S	S			X
27	34P	ESP	5/11/2005	PA	i	MP I /IP R	S	S	CI S/ LE R	I	S	S	X		
30	40P	AT	7/1/2013	PA	i	S	S	S	S	S	S	S	X		

ID pt: patient identification; ID: strain code; SAM: Sample; Date: Date of collection; Str: Strain; Ph: Phenotype; CAR: Carbapenems; MP: Meropenem; IP: Imipenem; PTC: Piperacillin/tazobactam; AM: Aminoglycosides; QUIN: Quinolones; CI: Ciprofloxacin; LE: Levofloxacin; MB: Monobactam; CEF: Cephalosporins; COL: Colistin; 1 St: *P. aeruginosa* first isolate; E: *P. aeruginosa* early isolate; L: *P. aeruginosa* late isolate; Esp: sputum; AT: hypopharyngeal suction; TF: throat swabs; PA: *Pseudomonas aeruginosa*; PA MDR: *P. aeruginosa* multi-drug resistant; PA MBL+: *P. aeruginosa* Metallo-Beta-Lactamases producing; s: small colony phenotype; w: wrinkled colony surface; m: mucoid colony; i: irregular colony edges; sm: smooth phenotype; R: Resistant; S: Susceptible; I: Intermediate; X: denotes positive for the feature.
